# Should Psychiatry Be Consulted When Facing a Self-Inflicted Foreign Body in the Urinary Tract?

**DOI:** 10.7759/cureus.23400

**Published:** 2022-03-22

**Authors:** Juan Carlos Angulo-Lozano, Nezahualcoyotl Gonzaga-Carlos, Maria F Virgen-Rivera, Luisa Fernanda Sanchez-Musi, Maria Jose Acosta-Falomir, Roberto De la Cruz-Galvan, Irene A Castillo-Del Toro, Jorge E Magaña-Gonzalez, Francisco Virgen-Gutierrez, Jorge Jaspersen Gastelum

**Affiliations:** 1 School of Medicine, Universidad Anáhuac Mexico, Huixquilucan, MEX; 2 Department of Urology, Hospital General de Mexico, Mexico City, MEX; 3 Department of Radiology, The American British Cowdray Medical Center, Mexico City, MEX; 4 Department of Psychiatry, Hospital General de Mexico, Mexico City, MEX

**Keywords:** sexual behavior, emergency, paraphilias, foreign bodies in genitals, foreign bodies in urinary tract

## Abstract

Background

On encountering a self-inflicted foreign body in the urinary tract, it is common that emergency physicians only consult the department of urology, and no further evaluations from other specialties are sought. Psychological conditions can also involve people with psychiatric disorders who perform self-harming or sexual practices. Many case reports of foreign bodies have been reported in the literature. However, there is little information regarding which specialties to consult in this situation within the emergency department (ED).

Methodology

This case series study gathered information on 10 cases from patients who attended the ED from 2005 to 2020 with the diagnosis of genital or lower urinary tract foreign body.

Results

In total, 10 patients were analyzed with a mean age of 37.3 (SD: ±14.1) years. Of the 10 patients, seven (70%) were males, and three (30%) were females. Overall, four (40%) patients presented with lower urinary tract symptoms (dysuria, tenesmus, hematuria, urinary frequency), five (50%) patients had a significant psychiatric history, and eight (80%) patients admitted having these practices for sexual gratification.

Conclusions

Foreign bodies in the lower urinary tract pose a significant challenge to ED physicians and urologists because some patients do not admit or do not recall inserting foreign bodies. Patients should be interrogated for mental illness, medication use, and a history of foreign bodies in the urinary tract or genitals during the initial evaluation. There is no consensus or screening method for such patients presenting to the ED. Hence, the use of complementary imaging studies and cystoscopy is fundamental for diagnosis. Further, it is essential to perform a psychiatric evaluation to diagnose or address any underlying psychiatric conditions that could cause this behavior.

## Introduction

Although foreign bodies in the urinary tract are not common in urologic emergencies, in the medical literature, several studies have been published as isolated cases or complications of introducing foreign bodies into the lower urinary tract. The most frequent cause of self-introduction of foreign bodies is for erotic purposes, but it is not the only cause [1,2]. The migration of objects toward the bladder cavity has also been described. The clinical presentation of such cases is diverse, generally associated with obstructive and irritative symptoms as well as severe complications. This is due to the delay in seeking medical attention because of the stigma and fear associated with the ethical and moral implications of the introduction of foreign objects and the pathology implicitly carried [3-5]. The diagnosis can be accidental while performing an imaging test, such as a simple X-ray or an abdominal ultrasound. It may be more complicated when it is not accidental due to the person’s non-recognition of the act or the impossibility of suspecting it [5,6]. Therefore, on many occasions, the diagnosis is delayed, even for years, with the patient presenting with prolonged symptoms that do not disappear despite therapeutic maneuvers [7].

Psychological conditions may also include individuals with psychiatric disorders, such as intellectual disability or schizophrenia, who perform self-harming or sexual practices [8-10]. Several diverse objects and triggering circumstances have been reported and broadly classified into two groups, namely, involuntary or voluntary introduction [9]. Individuals who voluntarily insert objects through the urethra exhibit paraphilic attitudes [10]. Paraphilias are sexual behavioral disorders that can affect many areas, including urology [11]. Paraphilias (from the Greek para meaning “parallel” or “opposite” and philia meaning “love” or “pleasure”) are stimuli or acts considered as deviations from normal sexual behavior (considering the term normal statistically as the most frequent) [12]. Although most people occasionally experience transient paraphilic behaviors, some individuals need only and exclusively these behaviors to continue to experience arousal and reach orgasm, with inhibited responses to typical erotic stimuli [11,12]. Urological paraphilias are also referred to as urophilia [13,14]. This includes pleasure through habits related to the urological sphere. While encountering intravesical foreign bodies, the initial approach should be endoscopic. Endoscopes have been described due to their larger caliber and the possibility of extracting larger objects [15]. Open bladder surgery is considered to be the last resort in large foreign bodies or significant associated injuries [16]. Once inside the bladder, all foreign bodies tend to be covered with phosphate and calcium salts [15,16], depending on the pH, the concentration of the urine, and the degree of infection [7]. Over time, these bodies can turn into large stones, as in the cases presented in this study. This study evaluates 10 patients and describes the dominant etiology of sexual practices or self-harming activities.

## Materials and methods

Patient selection

This case series collected information on 10 patients who attended the emergency department (ED) from 2005 to 2020. Inclusion criteria included patients who underwent a psychiatric evaluation by a psychiatrist (Hamilton test for depression and anxiety, Patient Health Questionnaire (PHQ) tests for teenagers, direct interrogation of sexual behaviors and practices at admission, and those who had a foreign body in the lower urinary tract (urethral meatus, urethra, or bladder). Patients who did not undergo a Hamilton test for depression and anxiety and were not directly interrogated for sexual behavior were excluded from the study.

Statistical analysis

Age was reported as mean and standard deviation using Minitab statistical software. The categorical variables such as sex, lower urinary tract symptoms, psychiatric history, sexual gratification, involuntary introduction, complications, and the time of foreign body in the urinary tract were reported in frequencies and percentages. Type of foreign body, site of foreign body, and treatment were reported in a table.

Case presentation

Relevant information for each patient was described, such as chronic disease, medication use, psychiatric illness, symptoms at admission, management, outcomes, and complications. Only the most relevant figures of the cases are included in this study. Computed tomography (CT) scans and radiograph images were interpreted by a board-certified radiologist with a magnetic resonance imaging (MRI) fellowship.

## Results

In total, 10 patients were analyzed with a mean age of 37.3 (SD: ±14.1) years. Descriptions of patient characteristics, foreign bodies, and treatment are summarized in Table 1.

**Table 1:** Summary of the cases and characteristics. M: male; F: female

Case number	Age	Sex	Type of foreign body	Site of foreign body	Circumstance of entry	Treatment
1	48	M	Metallic nut	Bladder	Self-inflicted	Cystolithotomy
2	57	M	Toothbrush	Rectum and bladder	Self-inflicted	Colostomy and cystostomy
3	42	M	Five silicone bars	Urethra and bladder	Self-inflicted	Cystoscopy with the extraction of the bars
4	21	F	Wooden pencil	Urethra	Self-inflicted	Cystoscopy with the extraction of the wooden pencil
5	43	M	50 cm long plastic cord	Urethra and bladder	Self-inflicted	Cystostomy
6	25	M	Wooden pencil	Urethra	Self-inflicted	Total extraction of the wooden pencil
7	53	M	Metal bearing	Penis	Self-inflicted	Circular saw cut
8	38	F	Eyeliner	Urethra	Self-inflicted	Cystoscopy with the extraction of the eyeliner
9	14	M	Seven beans	Urethra and Bladder	Self-inflicted	Cystoscopy with the extraction of the beans
10	32	F	Contraceptive ring	Bladder	Self-inflicted	Cystoscopy with extraction

In total, seven (70%) patients were male and three (30%) were female. Four (40%) presented with lower urinary tract symptoms (dysuria, tenesmus, hematuria, urinary frequency), five (50%) had a significant psychiatric history, eight (80%) admitted performing these activities for sexual gratification, and two (20%) patients introduced foreign bodies incidentally or did not remember introducing foreign objects in their urinary tract. Overall, 10 (100%) injuries were self-inflicted, one (10%) developed a recto-vesical fistula, and three (30%) had a foreign body in the bladder for more than one month (Table 2).

**Table 2:** Frequencies of foreign body risk factors, behavioral characteristics, and symptoms.

Characteristics	Frequency
Female sex	3 (30%)
Male sex	7 (70%)
Lower urinary tract symptoms	4 (40%)
Psychiatric history	5 (50%)
Sexual gratification	8 (80%)
Involuntary introduction	2 (20%)
Self-inflicted	10 (100%)
Complications	1 (10%)
Foreign body for more than one month in the urinary tract.	3 (30%)

Case 1

**Figure 1:**
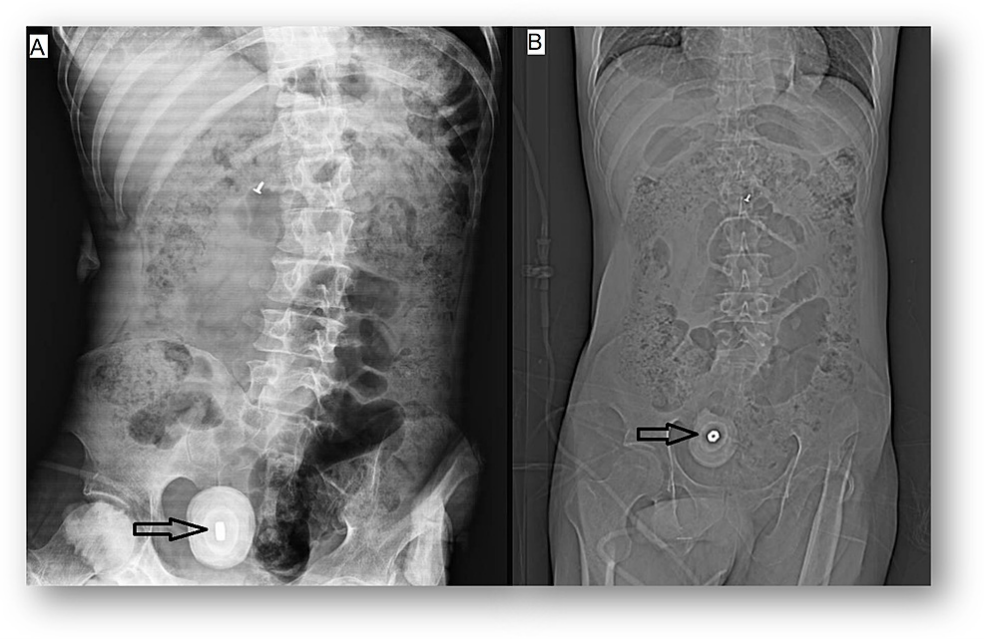
(A) Abdomen Rx with a radiopaque oval lesion on the pelvis (black arrow). (B) CT of the abdomen scout with an oval lesion with metallic density on its center (black arrow). Rx: radiography; CT: computed tomography

**Figure 2:**
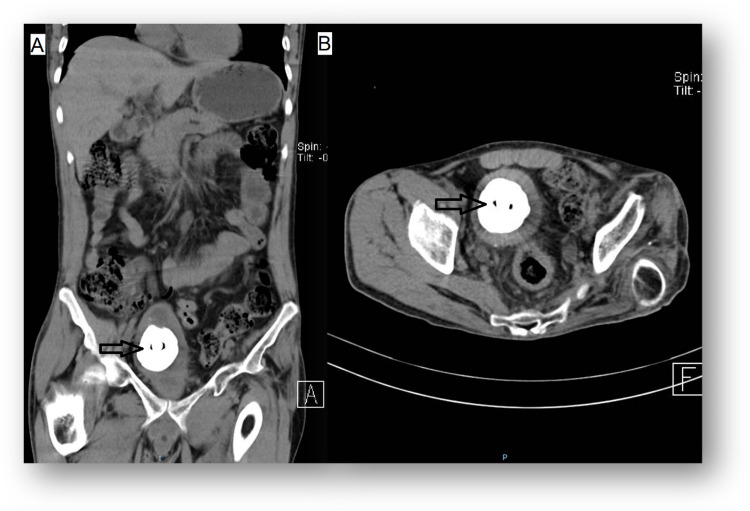
A foreign body with metal density (black arrow) in the center of the oval image in the bladder with a hyperdense capsule surrounding it forming a bladder stone measuring 6 × 7 cm in coronal (A) and axial (B) views.

A 48-year-old male presented with a 16-year history of controlled schizophrenia with risperidone 3 mg once daily, alprazolam 0.25 mg once daily, and biperiden 2 mg once daily. He had recurrent urinary tract infections for 18 months and was treated with multiple antibiotic regimens without improvement. He presented to the emergency department with fever, hypotension, and tachycardia. The patient was admitted and diagnosed with urosepsis. Further imaging studies were performed (Figures 1, 2).

He denied inserting a foreign body into his genitals. The psychiatry department was consulted. He had been without positive or negative symptoms for over a year. The positive and Negative Syndrome Scale was rated at 1, and the Scale for Assessment of Negative Symptoms was rated at 1. No adjustments were made to the treatment.

Case 2

**Figure 3:**
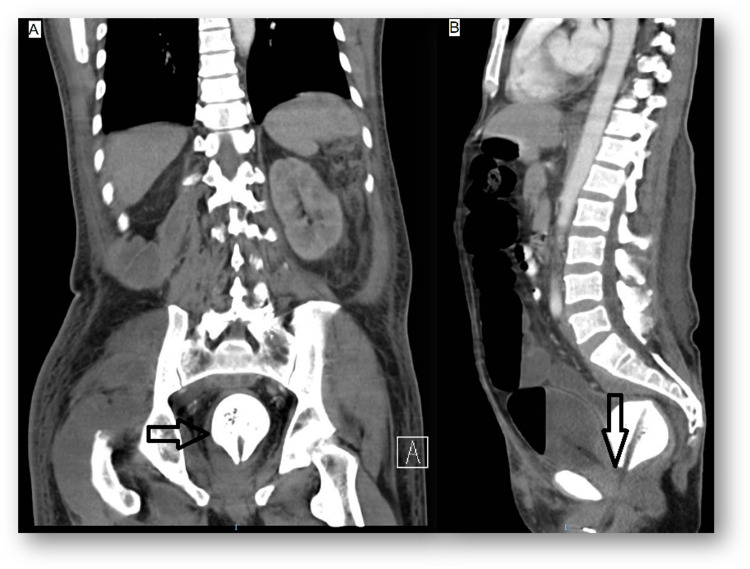
CT scan of abdomen and pelvis showing an acute inflammatory process (black arrow) with a pneumatic component, Foley catheter, and hyperdense artifact that conditions rectovesical fistula on coronary (A) and sagittal (B) planes. CT: computed tomography

A 57-year-old male with a history of congenital neurosensorial hypoacusis was brought to the ED with a 20-day evolution of intermittent fever, dysuria, urinary frequency, pneumaturia, and fetid urine. An abdominal CT scan was performed (Figure 3).

On physical examination, there was the passage of urine through the anus. Exploratory laparotomy was performed. A foreign body was noted in the rectum, consistent with a toothbrush with calcified tufts, communicating with the posterior wall of the bladder and forming a bladder stone of 5 × 5 cm. Further colostomy and cystostomy were performed. No complications presented after surgery and the patient was discharged. Major depressive disorder was diagnosed, and he was prescribed sertraline 50 mg with follow-up every month.

Case 3

A 42-year-old man with a 10-year history of active major depressive disorder treated with fluoxetine with poor adherence and heavy alcohol consumption presented to the ED 48 hours after introducing five silicone bars through the urethra while he was drunk. He reported urinary frequency, dysuria, and macroscopic hematuria during urination. A cystoscopy was performed reporting an edematous and erythematous urethra, with the integrity of the urethral mucosa and external sphincter; at this level, the first silicone bar was observed and extracted. The bladder had hemorrhagic traces in the mucosa and pseudodiverticula, and the cavity was occupied by multiple silicone bars. All of them were removed successfully with no complications. Hamilton Rating Scale for Depression was 18, and he was diagnosed with moderate depression. Postoperative findings were nine silicone bars. He was discharged with no further complications 24 hours after the procedure. Follow-up one week later showed remission of symptoms.

Case 4

A 21-year-old female without any relevant history came to the ED because of macroscopic hematuria without clots and irritative lower urinary tract symptoms. She admitted introducing a wooden pencil through her urethra hours before. Cystoscopy showed edematous urethral mucosa with evidence of urethral tearing, with hemorrhagic traces and the presence of a wooden pencil that was successfully extracted. Evaluation for anxiety and depression using the Hamilton Rating Scale was normal, and no other paraphilias were diagnosed on interrogation.

Case 5

**Figure 4:**
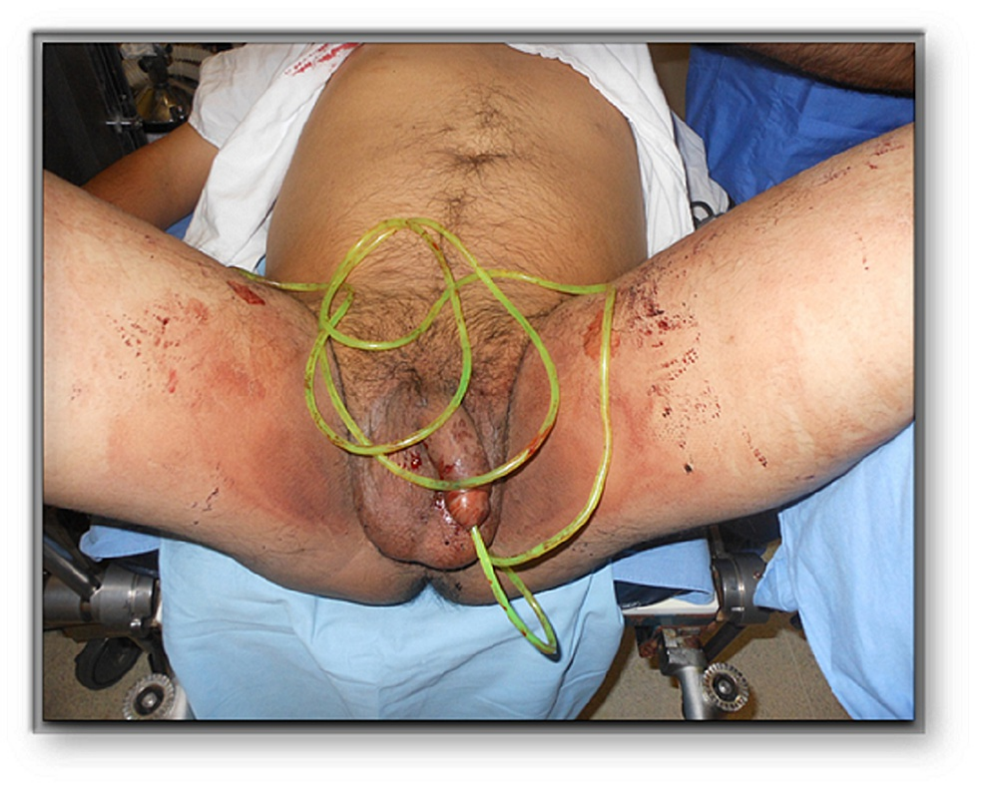
Plastic cord introduced in the meatus of a middle-aged male.

A 43-year-old man with a history of introducing foreign bodies in the urethra and major depression disorder without treatment came to the ED with acute urinary retention and urethrorrhagia after introducing a 50 cm long plastic cord through his urinary meatus (Figure 4).

He said that the cord got stuck in this urinary tract, following which he sought medical attention. The patient was admitted and part of the plastic cord was removed manually with lubrication. Then a cystoscopy was performed and showed an intravesical portion of the cable folded with the inability to extract the intravesical part. Total extraction of the intravesical portion was successfully done by cystostomy. Hamilton test for anxiety and depression was performed by a psychiatrist in the ED and the scores were negative for mental illness. No other symptoms were present. He was referred to psychology for cognitive-behavioral therapy.

Case 6

**Figure 5:**
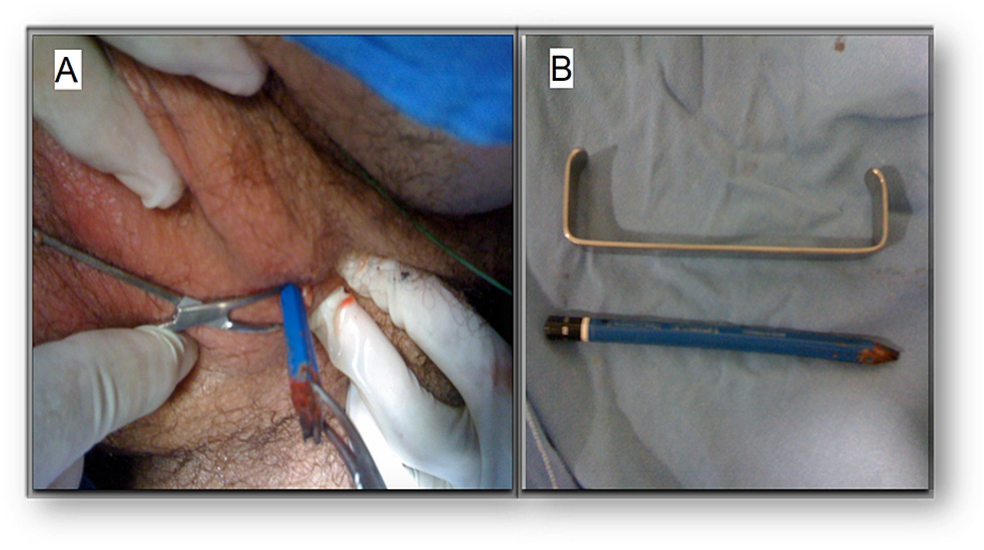
The wooden pencil stuck in the patient’s perineum (A), pencil compared with a Farabeuf retractor after total extraction (B).

A 25-year-old man with a history of brief psychotic disorder with no medications and no other relevant medical history was brought by his mother to the ED because of severe pain and perineal inflammation for three hours. During medical interrogation, the patient confessed that he introduced a wooden pencil through his urethra to obtain sexual gratification and could not extract the pencil. On physical examination, erythema and inflammation of the perineal region were observed with a fistulous orifice through which the pencil was observed, as shown in Figure 5.

Total extraction of the pencil by traction was successful. Subsequently, a suprapubic catheter was placed to shunt the urinary tract. He presented with urethral stenosis six weeks after, and urethral reconstruction was performed. A psychiatric evaluation was normal.

Case 7

A 53-year-old man with a history of controlled hypertension and no other significant history presented to the ED due to edema of his penis and acute urinary retention. The patient stated that he introduced his penis in a metal-bearing to maintain a longer-lasting erection. After several failed attempts to remove the metal-bearing, he came to get medical attention at a hospital. The metal-bearing was cut with a circular saw in the operating room and removed without complications. Psychiatric evaluation was normal. He was discharged without complications.

Case 8

A 38-year-old female with no significant history came to the ED because an eyeliner got stuck in her urethra while masturbating. She said that she had been doing it for five years without complications. She did not have hematuria or irritative lower urinary tract symptoms. Cystoscopy was performed, and visualization of a foreign body compatible with eyeliner was observed. The eyeliner was removed with no complications. The patient was discharged with oral antibiotics. Psychiatry referred the patient to the psychology department for cognitive-behavioral therapy, and no medication was indicated.

Case 9

A 14-year-old boy diagnosed with attention-deficit hyperactivity disorder (ADHD) two years ago being treated with methylphenidate with no other relevant history was brought to the ED by his mother for acute urinary retention. On private interrogation, the boy said he introduced seven beans in his urinary meatus, hoping to get sexual satisfaction 12 hours before coming to the hospital. Cystoscopy was performed and showed edematous urethra with urethral beans and intravesical beans, which were removed without any complications with a total of seven beans. Department of Psychiatry was consulted and a psychiatrist evaluated the patient in the ED. PHQ-9 was negative for depression and PHQ-4 was negative for anxiety. The patient had a psychiatry follow-up for his ADHD. A higher dose of methylphenidate was prescribed from 10 mg to 36 mg.

Case 10

**Figure 6:**
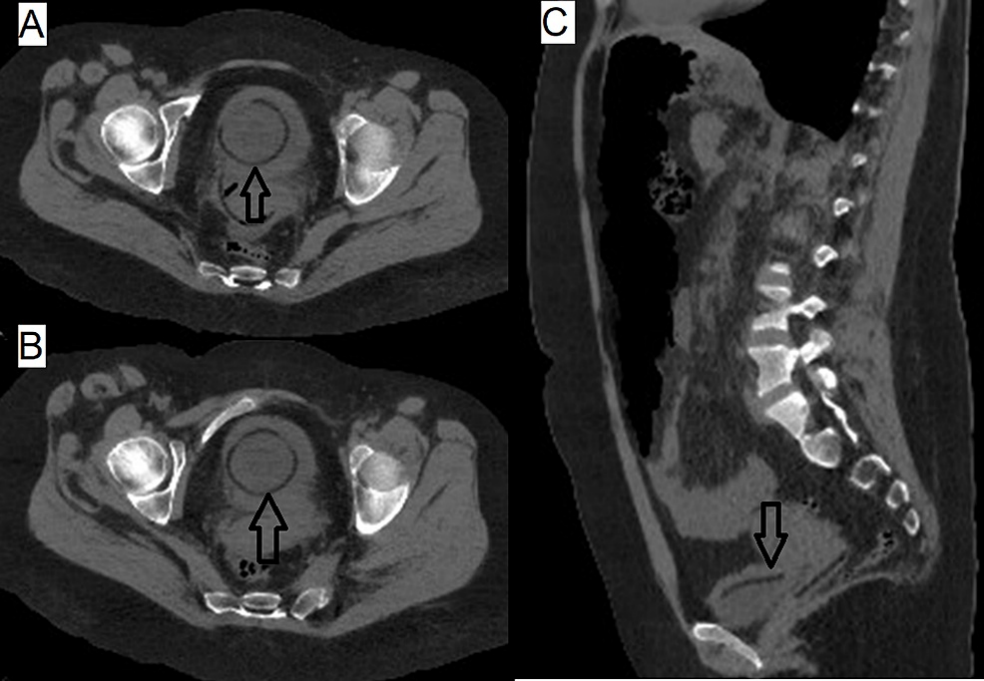
Axial (A and B) and sagittal (C) views show two round hypodense foreign bodies, one within the bladder pointed by the black arrows and the other one in the vaginal canal.

A 32-year-old female presented to the ED after a referral from a primary care physician because of persistent lower urinary tract symptoms with no improvement after three prescriptions of different antibiotics in two months. She had no relevant family or personal medical history. Diffuse abdominal tenderness was present with significant pain in the suprapubic region on physical examination. The temperature was 38.2°C, and the rest of the vital signs were within the normal range. CT scan of abdomen and pelvis was performed, as shown in Figure 6.

Urology was consulted, and intravenous amikacin was prescribed. Cystoscopy with the extraction of the foreign body was performed. A hormonal vaginal ring was extracted. There were no complications after the procedure. Psychiatry evaluation was negative for paraphilias, anxiety, or depression. She was discharged with levofloxacin 1 g per day for 14 days. Symptoms resolved with negative urine cultures, and she was discharged.

## Discussion

Foreign bodies can present as insidious or recurrent urinary tract infections despite appropriate management, as seen in some of these cases. Schizophrenia, major depressive disorder, and generalized anxiety disorder should be interrogated at admission. Acute urinary retention and hematuria might be present on examination and should raise suspicion [17]. Previous studies have reported a male preponderance in urinary foreign bodies, as seen in this study [18]. In this study, half of the patients had a psychiatric history, more than the average reported in previous studies. One reason for these findings could be a result of not asking if patients had a psychiatric history and not evaluating further. Sexual gratification was the main reason for the introduction of foreign bodies. According to Gooren et al., these conducts tend to be predominantly driven by compulsions rather than sexual conduct [11]. The patients in this study did not receive pharmacological treatment for paraphilias because none demonstrated efficacy or indicated such conduct [11]; however, they were referred to psychological therapy. Classification of foreign bodies in the urinary tract is shown in Table 3 [1,18,19].

**Table 3:** List of foreign bodies reported in the bladder.

Reported objects	
Organic materials	Animal parts (dog penis, ants, snails, squirrel spine). Plants and vegetables (grass, pieces of wood, elm, beans). Viscous liquids or substances (molten paraffin, glue, chewing gum)
Sharp and wire-type objects	Pencils, hairpins, pens, needles, plastic tubes, catheters, various types of electrical wires, metal wires, lines
Atypical objects	Pearl buttons, adhesive tape, tampon, chewing gum, large plastic bars, ink cartridge
Objects that reach the bladder via traumatic injury	Bullets, pieces of patient clothing, bone chips, wood
Iatrogenic foreign bodies	Urinary catheter tips, catheter balloon parts, or endoscope material, as well as staples, sponges, gauze, swabs, umbilical tape, bone wax, surgical glove fragments, IUDs, pessaries
Migrated foreign bodies	Gastrointestinal tract: chicken and fish bones, pins, needles, pencils, thermometers, ingested bullets, mold teeth, tobacco pipe mouthpiece. Female genital tract: perforating dermoid cyst hairs of the ovary, skeletal remnants of extrauterine pregnancies. Coming from vaginal perforations: cucumbers, hairpins, wooden last
Others	Hair, broom straws, perfume bottles, toothbrush

Anecdotally, a few articles address the epidemiology of foreign bodies in the urinary tract and mental pathology. Rodríguez et al. reported that female patients with mental illness tend to introduce foreign bodies in the urethra and bladder more often than in the vagina. According to this study, 23.6% of the patients studied had a mental illness, with mood disorders being the most common. Mental illness, such as schizophrenia and mood problems, affected 44% of the men in this study [19]. Self-insertion of a foreign body is commonly noted in patients with persistent psychosis for self-harm or eroticism, according to Ahsaini et al. [20]. In rare situations, it is done for self-mutilation in patients with schizophrenia, as one patient in this study had a history of schizophrenia [20]. Urologists must report these patients to a psychiatric consultation to discover and treat any probable mental disorder and avoid any future acts of self-mutilation [20]. Chouaib et al. reported a patient with a psychiatric evaluation requested by the ED with a history of urethral sounding, which consists of introducing objects in the urethra for sexual gratification, who was diagnosed with major depressive disorder and recurrent suicidal thoughts, requiring urgent attention with involuntary admission after surgery [21]. Sukkarieh et al. described the main etiologies and reported the conducts that explained the introduction of objects in the urethra. They reported that erotic urges, mental illnesses with a predominance among patients with personality disorders, and intoxication with psychoactive substances were predominant [22]. In 18 patients studied for introducing foreign bodies, six admitted doing it for sexual gratification (33%) in contrast to the 80% reported in our study, seven for therapeutic purposes (urination) (39%), and two for psychiatric illness (11%). In three patients, no definite reason was reported (17%) [23]. Shiah et al. proposed using topiramate at a dose of 200 mg daily to treat paraphilias in one patient, with success and no reported side effects [24]. Wan et al. reported another successful case of a patient with schizophrenia and paraphilic disorders treated with topiramate; further studies on the effectiveness of topiramate are required to confirm its efficacy on this patient population [25]. Previous studies have attempted to explain paraphilias from an endocrinological perspective. There is controversy when diagnosing paraphilias as some authors predict that paraphilias are a cultural phenomenon and not a psychiatric condition [26]. The Diagnostic and Statistical Manual of Mental Disorders, Fifth Edition (DSM-V) differentiates between atypical sexual interest and mental disorder patients who exhibit personal distress about their interest despite social disapproval or a sexual desire that involves psychological distress from another person, injury, or death. Under the latest classification of paraphilias in DSM-V, urophilia is considered as “other” specified paraphilic disorder [14]. A previous study suggested the removal of paraphilias from DSM arguing that functioning paraphilias may be impaired because sexual interest is a mental illness, concluding that the definition of paraphilia is the cause of the dysfunction [27]. There have been small studies and case reports of pharmacological treatment for non-criminal paraphilias, but there is no conclusive evidence supporting the rational use of some anticonvulsants and antipsychotics for the treatment of this disorder. A major problem is that the majority of the paraphilias are negligible, and it depends on voluntary disclosure, which is very rare, with the justification of treatment remaining controversial [27]. Similar to this study, patients sought medical attention because of a complication in their sexual behavior and not because they wanted to change their behaviors. Beech et al. concluded that behavioral therapy such as aversion and reconditioning had no evidence of therapeutic efficacy [28]. The results of this study should be interpreted with caution because the study population was limited. Based on the results, we suggest consulting psychiatry when facing a self-inflicted foreign body for a complete evaluation.

## Conclusions

Foreign bodies in the lower urinary tract pose a significant challenge to ED physicians and urologists because some patients do not admit or do not recall inserting foreign bodies. Patients should be interrogated for mental illness, medication use, and a history of foreign bodies in the urinary tract or genitals during the initial evaluation. There is no consensus or screening method for this patient population in the ED. Therefore, the use of complementary imaging studies and cystoscopy is fundamental for diagnosis. It is essential to perform a psychiatric evaluation to diagnose or address any underlying psychiatric conditions that can cause this behavior. Categorizing normal versus deviant or disordered sexuality is the biggest challenge when using the term paraphilia as these definitions have been highly controversial and debatable. Many patients are discharged when the urology team resolves the problem without further assessment. There is a risk of urinary complications from repetitive harmful sexual behaviors. Urethral stenosis and strictures are expected consequences of these practices, but most patients have good outcomes and quality of life. We suggest multidisciplinary management for these patients with urology and psychiatry consultation when the emergency room doctor encounters self-inflicted foreign bodies in the urinary tract as half of the patients in this study had a relevant psychiatric history.
